# The Zebrafish Retina and the Evolution of the Onecut-Mediated Pathway in Cell Type Differentiation

**DOI:** 10.3390/cells13242071

**Published:** 2024-12-15

**Authors:** Quirino Attilio Vassalli, Giulia Fasano, Valeria Nittoli, Eleonora Gagliardi, Rosa Maria Sepe, Aldo Donizetti, Francesco Aniello, Paolo Sordino, Robert Kelsh, Annamaria Locascio

**Affiliations:** 1 Department of Biology and Evolution of Marine Organisms, Stazione Zoologica Anton Dohrn, 80121 Napoli, Italy; quirino.attilio@gmail.com (Q.A.V.); giulia.fasano@opbg.net (G.F.); valeria.nittoli@biogem.it (V.N.); eg228@st-andrews.ac.uk (E.G.); rosamaria.sepe@szn.it (R.M.S.); 2 Department of Biology, University of Naples Federico II, 80126 Naples, Italy; aldo.donizetti@unina.it (A.D.); faniello@unina.it (F.A.); 3 Department of Biology and Evolution of Marine Organisms, Sicily Marine Centre, Stazione Zoologica Anton Dohrn, 98167 Messina, Italy; paolo.sordino@szn.it; 4 Department of Life Sciences, University of Bath, Bath BA2 7AY, UK; bssrnk@bath.ac.uk

**Keywords:** *Danio rerio*, eye, *Hnf6*, genetic pathway, comparative evolution, photoreceptor

## Abstract

*Onecut/Hnf6* (*Oc*) genes play an important role in the proper formation of retinal cells in vertebrates, in particular horizontal, retinal ganglion and amacrine cells. However, it is not fully known how the unique and combined action of multiple *Oc* gene copies leads to the induction and differentiation of specific retinal cell types. To gain new insights on how *Oc* genes influence retina formation, we have examined the developmental role of *oc1*, *oc2* and *oc-like* genes during eye formation in the non-mammalian vertebrate zebrafish *Danio rerio*. By using single and multiple morpholino knockdown of three zebrafish *Oc* genes we provide evidence for the independent and redundant role of each gene in the formation of photoreceptors and other retinal tissues. Through comparison of *Oc* genetic pathways in photoreceptor differentiation among chordates we demonstrate their mechanism of action through a series of conserved target genes involved in neural transmission.

## 1. Introduction

Neural cells are among the most diversified functional units of an organism and make a critical contribution to the high complexity of the nervous system. An interesting example of highly diversified nerve cells is those that make up the vertebrate eye. These retinal cells represent an excellent model for investigating how light perception, light direction recognition and high-resolution imaging formation evolved [1]. In particular, the genetic control of photoreceptor cell (PRC) development needs to be precisely regulated in order to give rise to functional cells capable of converting light into nerve impulses and conveying them to the brain [2]. Great progress in understanding the transcriptional network that controls PRC specification and development has been made over the last 20 years. Nevertheless, our comprehension of these molecular mechanisms still contains many gaps. In this context, it has recently become clear that *Onecut/Hnf6* (*Oc*) genes play a fundamental role in regulating the specification and differentiation of different neural cell types and, in particular, in the formation of various regions of the vertebrate retina [3].

From invertebrates to vertebrates, *Oc* genes encode for transcription factors implicated in multiple processes during development and differentiation [3]. Looking at the functional role of the single *Oc* gene in invertebrates [4,5,6,7,8] and its multiple copies in vertebrates, it is evident that these genes have a common and evolutionarily conserved role in the specification and differentiation of various neuronal cell types, and, in particular, in the formation of the diverse light sensing nerve cells (reviewed in [7]). In recent years, a conserved function of *Oc* in photoreceptor differentiation has been highlighted and investigated [9]. This role was originally identified in *Drosophila*, where *D-Onecut* induces the expression of the *rhodopsin* gene during the final stages of PRC differentiation [8]. Likewise, during chordate evolution, the single *Oc* gene of tunicates plays a primary role in PRC differentiation [9]. Vertebrates contain three to five *Oc* genes, and the duplication of these genes is associated with the acquisition of more specialized roles, not only in the formation of the various neural tissues but also in the development and differentiation of liver and pancreatic cell types [3,7,10].

**Figure 1:**
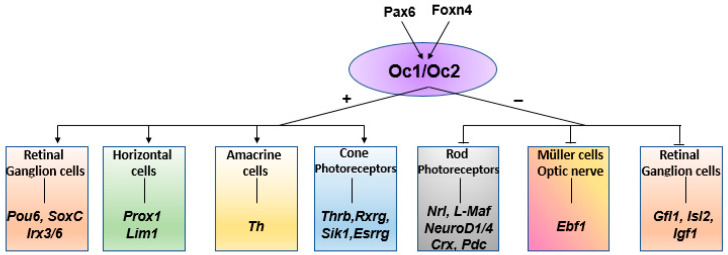
Onecut genetic cascade in the vertebrate retina. Schematic representation of the *oc1* and *oc2* vertebrate transcription factors, their upstream regulators and their target genes involved in the development of retinal tissues.

In recent years, there has been increasing interest in understanding the genetic pathways that enable these factors to regulate the differentiation of vertebrate retinal cell types (Figure 1). Among the three *Oc* genes identified in mice, different combinations of *Oc1* and *Oc2* in retinal progenitor cells (RPCs) have been shown to control the fate of horizontal cells (HCs), amacrine cells (ACs) and cone PRCs. *Oc1* gene expression begins early in retinal development, in precursors of retinal ganglion cells (RGCs), HCs and cone PRCs [11,12,13,14,15,16]. The simultaneous action of *Oc1* and *Otx2* promotes the fates of cone PRCs and HCs while repressing rod PRC differentiation [17].

In mice the cooperation of Pax6 and Foxn4 controls HC genesis, partly through the activation of *Oc* genes (Figure 1). Foxn4 stimulates the expression of *Oc1* and *Oc2* [15] and activates the expression of *Ptf1a* [17,18,19]. Subsequently, Ptf1A is required for the regulation of *Lim1* and *Prox1* during early HC development, while in the mature retina, *Oc1* and *Oc2* are expressed exclusively in HCs, where they regulate their maintenance [15,16]. The combined action of *Oc1* and *Oc2* leads to the activation and repression of a specific set of genes necessary for promoting RPC differentiation [15,16](Figure 1). The combined deletion of these two factors results in a complete loss of HCs and the absence of the outer plexiform layer (OPL) [7,11,12,13]. Furthermore, their knockdown also leads to age-dependent PRC degeneration due to the absence of HCs, suggesting an indirect role of *Oc* genes in PRC maintenance [11,16]. Later in retinal differentiation, *Oc1* and *Oc2* promote cone differentiation from PRC precursors while repressing rod differentiation (Figure 1) [11,12,17,20,21].

These two mouse *Oc* genes share common functions and target genes essential for the proper formation and differentiation of retinal tissues [11]. Their simultaneous downregulation leads to a more severe retinal phenotype compared to that of the individual mutants [15,16]. Due to this redundancy, it has been challenging to precisely define the mechanisms underlying their retinal cell fate determination, and little information is available on the regulatory targets of each *Oc* gene during eye formation.

Among vertebrates, teleost fish show additional duplications of *oc* genes, resulting in the formation of an *oc-like* and two *oc3* genes (*oc3a* and *oc3b*). Despite their branch-specific gene duplications, teleost fish represent a valuable model for investigating the evolution of genetic pathways from invertebrate to vertebrate chordates and for uncovering both unique and common features of each *oc* gene. Furthermore, the teleost zebrafish *Danio rerio* is a promising model system for studying the effects of the combinatorial downregulation of *oc* genes. In this study, we provide information about the role of the three *oc* genes expressed in the zebrafish retina (*oc1*, *oc2*, *oc-like*) and their regulatory network controlling retinal tissue differentiation. Moreover, we compared the expression data for the downstream targets of *Oc* genes identified in ascidians [9,22] and mice [11], and investigated their involvement in zebrafish photoreceptor differentiation. Taken together, this study highlights the ancient assembly of the *Oc* genetic pathway in RPC cell differentiation and its conservation during chordate evolution.

## 2. Materials and Methods

### 2.1. Zebrafish Maintenance and Sample Collection

Zebrafish *D. rerio* maintenance and experiments were performed with the approval of the University of Bath ethics committee and in full accordance with the Animals (Scientific Procedures) Act 1986, under Home Office Project Licenses 30/2937. Fish were grown and housed in a recirculating water system under controlled husbandry conditions: 14 h light/10 h dark cycle, temperature 28 °C, conductivity 350–400 µS, pH 6.8–7.2 and fed daily [23]. Natural matings of adult fish pairs (AB, wild-type and SoFa1 line, Atoh7:gapRFP/Ptf1a:cytGFP/Crx:gapCFP) were conducted in appropriate mating tanks, and zebrafish embryos were collected and cultured in a Petri dish with embryo medium (E3 medium) in a dedicated incubator at 28 °C under a 14 h light/10 h dark lighting cycle at 28 °C. 

### 2.2. Identification of Zebrafish Orthologs of Ascidian Onecut Target Genes

To define the putative best homologs between the ascidian *Ciona robusta* and zebrafish *Danio rerio*, a BLASTx (e-value: 0.001) was performed, aligning the *Ciona* transcripts versus the complete set of zebrafish proteins (Fasta version GRCz11, downloaded from the NCBI at https://www.ncbi.nlm.nih.gov/assembly/GCF_000002035.6 (accessed on 4 August 2024), version of November 2016).

The orthologs between *Ciona* and mice were identified by performing (i) a tBLASTx (e-value: 0.001) of *Ciona* transcripts versus mouse transcripts; (ii) a tBLASTx (e-value: 0.001) of mouse transcripts versus *Ciona* transcripts and (iii) the Transcriptologs approach (80) to find the bidirectional best hits (BBHs). Orthologous and paralogous genes were organized into gene networks, according to [24].

### 2.3. Whole Mount In Situ Hybridization (WISH) and Vibratome Tissue Sectioning

ClustalW multiple alignment of the five zebrafish *oc* cDNA sequences was used to identify cDNA sequences specific for each *oc* gene. These regions were amplified by PCR on cDNA at 24 hpf and used to prepare antisense riboprobes, as described by [25] (primers are listed in Table S1). Zebrafish embryos at 48 and 72 hpf were anesthetized with tricaine MS-222 (Sigma-Aldrich, MI, USA), fixed by immersion in 4% paraformaldehyde (PFA)/PBS (pH 7.4) at 4 °C overnight (ON) and, after sequentially washing through graded methanol scale (25%, 50%, 75% and 100%), stored in methanol 100% at −20 °C. WISH for *oc1*, *oc2*, *oc-like* and *oc3* (*oc3a* and *oc3b*) paralogs was performed on 20 zebrafish embryos for each gene as described by [26]. After WISH, zebrafish embryos were transferred to a plastic mold and embedded according to the desired sectioning plane in a mix of BSA and gelatin and (Sigma-Aldrich, MI, USA) and processed for vibratome tissue cross-sectioning. Then, differential interference contrast (DIC) microscopy images were acquired with an Axio Imager M1 microscope equipped with an Axiocam digital camera (Zeiss, Oberkochen, Germany).

### 2.4. Morpholino-Mediated Knockdown

Morpholino (MO) antisense oligonucleotides directed against *oc1*, *oc2* and *oc-like* were obtained from GeneTools LLC. (Philomath, OR, USA) and MO sequences listed in Table S1. MO specificity was assessed by blasting all *oc* MO sequences against a GRCz11 reference assembly genome and by alignment of *oc1*, *oc2* and *oc-like* 5’UTR/exon 1 sequences. Different amounts of MOs were microinjected in one-cell stage embryos and the embryo survival rate of *oc1, oc2* and *oc-like* morphant embryos at 24 hpf was evaluated compared to control embryos microinjected with a standard (STD) MO targeting the human β-*globin* pre-mRNA (Figure S1) [27]. After performing a MO dose–response curve, *oc1, oc2* and *oc-like* MOs were microinjected at the following concentrations: 300 pg for *oc1* and 500 pg for both *oc2* and *oc-like* paralogs. Concomitant injection of half doses of each MO was used to generate *oc*-triple morphants (150 pg *oc1*, 250 pg *oc2*, 250 pg *oc-like*). MO rescue experiments were performed by injecting 300 pg of *oc1* MO and 200 pg of *oc1* mRNA.

### 2.5. Eye Size Measurement

*oc1*, *oc2*, *oc-like* and *oc*-triple zebrafish morphants were evaluated at 24 hpf for eye size starting from an image acquired with AxioImager M1 microscope equipped with an Axiocam digital camera (Zeiss, Oberkochen, Germany). Raw images were run on Fiji software 1.51g [28] and the eye perimeter was manually designed using a polygonal selection tool. Eye size was measured as the total area for all experimental groups; multiple comparisons were assessed comparing each MO experimental condition to controls (STD MO). We analyzed 20 eyes per group for each replicate, and data are expressed as mean ± SEM (n = 3 replicates). Statistically significant differences were assessed by the non-parametric Kruskal–Wallis test with Dunn’s post hoc correction.

### 2.6. TUNEL Staining

Zebrafish embryos at 72 hpf were anesthetized with tricaine MS-222 (Sigma-Aldrich), fixed by immersion in 4% PFA/PBS at 4 °C ON and, after sequentially washing through graded ethanol scale (25%, 50%, 75% and 100%), stored in 100% ethanol at −20 °C. Fixed embryos were processed for the DeadEnd (TM) Colorimetric TUNEL System assay according to the manufacturer’s instructions (Promega, WI, USA). Briefly, zebrafish embryos were rehydrated by sequentially washing through graded ethanol washes (95%, 85%, 70% and 50%) for 3 min and incubating in 0.85% NaCl for 5 min at room temperature (RT). After washing in PBS and immersing in 4% paraformaldehyde for 15 min at RT, zebrafish embryos were permeabilized with 20 μg/mL Proteinase K for 30 min at RT. Samples were equilibrated in the equilibration buffer for 10 min at RT and incubated in freshly prepared rTdT Reaction Mix for 1 h at 37 °C. After several washes in PBS, zebrafish embryos were incubated in 0.3% hydrogen peroxide/PBS solution for 5 min and incubated in Streptavidin HRP solution/PBS (1:500). Samples were washed several times in PBS and incubated in DAB solution (DAB Chromogen + DAB Substrate) for at least 10 min until staining was developed. To block the staining, zebrafish embryos were rinsed several times in deionized water. For microscopy imaging, zebrafish embryos were laterally mounted and imaged on a AxioImager M1 microscope equipped with an Axiocam digital camera (Zeiss, Oberkochen, Germany). We analyzed 20 larvae per group for each replicate, and data were expressed as mean ± SEM (n = 3 replicates). Statistically significant differences were assessed by one-way ANOVA with Dunnett’s post hoc correction.

### 2.7. Total RNA Extraction, cDNA Synthesis and Quantitative Real Time PCR (RT-qPCR) Analysis

Zebrafish embryos at 48 hpf were anesthetized with tricaine MS-222 (Sigma-Aldrich, MI, USA) and dry stored at −80 °C. We collected 20 embryos per group for each replicate, the total RNA was isolated from whole embryonic tissue samples using TRIzol reagent (Invitrogen, MA, USA), and the quality and concentration were determined with the Agilent 2100 Bioanalyzer (Agilent Technologies, CA, USA). The first-strand cDNA was synthesized using the SuperScript™ VILO™ cDNA Synthesis Kit (Thermo Fisher, MA, USA) according to the manufacturer’s protocol. Each RT-qPCR reaction was carried out as follows: 1 µL cDNA, 280 nM final concentration of each primer (primers are listed in Table S1) and SYBR green qPCR mastermix (Thermo Fisher, MA, USA) in a total volume of 10 µL per well. Ribosomal protein L13a (*rpl13a)* and elongation factor 1-alpha 1 (*elfa1)* were used as reference genes. RT-qPCR reactions were performed in triplicate using a ViiA7 ABI thermal cycler (Applied Biosystems, MA, USA) with the following thermal profile: 95 °C for 20″, 1 cycle for cDNA denaturation; 95 °C for 1″ and 60 °C for 20″, 40 cycles for amplification; 95 °C for 15″, 60 °C for 1′ and 95 °C for 15″, 1 cycle for melting curve analysis, to verify the presence of a single product. Each assay included a no-template control for each primer pair. Data analysis was performed using the comparative 2−ΔΔCt method and mRNA levels were expressed as a fold change of the mean relative to controls (STD MO). Data were expressed as the mean of duplicate ± SEM. Statistically significant differences were assessed by one-way ANOVA with Dunnett’s post hoc correction.

### 2.8. Cryostat Sectioning and imMUNOFLUORESCENCe

Zebrafish embryos to be processed for immunofluorescence at 72 hpf were grown in 0.003% 1-phenyl 2-thiourea (Sigma-Aldrich, MI, USA) from 22 hpf to 72 hpf and anesthetized with tricaine MS-222 (Sigma-Aldrich, MI, USA). Then, embryos were fixed by immersion in 4% PFA/PBS at 4 °C ON. After several washes in PBS, embryos were incubated in 30 % sucrose/PBS overnight at 4 °C and, then, processed for embedding frozen tissue in OCT compound (Tissue Tek, Miles Scientific, MA, USA). Briefly, each embryo was transferred in a plastic mold and mounted according to the desired plane in the OCT compound and sectioned to obtain 10–16 micrometer-thick transverse cryosections using the cryostat CM1800 (Leica Biosystems, IL, USA). Cryosections were immunostained for CD166 antigen homolog A (Neurolin) Zn-8 and Synaptic vesicle glycoprotein SV2 and single immunofluorescence was performed as described in [29]. Antibodies used in this study were as follows: primary antibodies Zn-8 (1:10, Developmental Studies Hybridoma Bank, DSHB) and SV2 (1:100, DSHB) and secondary antibody Goat anti-mouse Alexa fluor 488 (1:400, Life Technologies, Thermo Fisher, MA, USA). Nuclei were counterstained with 100 ng/mL DAPI (4′,6-diamidino-2-phenylindole, Sigma-Aldrich, MI, USA) or with ToPRO 3 (Life Technologies, Thermo Fisher, MA, USA). Immunostained cryosections were imaged using a LSM700 confocal microscope (Zeiss, Oberkochen, Germany). For microscope acquisition of images from SoFa1 transgenic line (Atoh7:gapRFP/Ptf1a:cytGFP/Crx:gapCFP), transgenic zebrafish embryos at 72 hpf were treated and cryosectioned as described above. We performed Zn-8 and SV2 immunostaining on sections from five samples per group for each replicate, and concerning SoFa1 morphants, we analyzed sections from six animals per group for each replicate.

### 2.9. Whole-Mount Immmunostaining

Zebrafish embryos at 72 hpf were anesthetized with tricaine MS-222 (Sigma-Aldrich, MI, USA) and fixed by immersion in 4% PFA/PBS (pH 7.4). For whole-mount immunostaining for the mitotic marker phosphorylated H3 (PH3), zebrafish embryos were processed as described in Verduzco et al. [30]. The primary and secondary antibodies used were anti-PH3 antibody (1:500, Millipore, Darmstadt, Germany) and anti-rabbit IgG biotinylated secondary antibody (1:200, Vector Laboratories, CA, USA), respectively. After several washes to remove nonspecific bindings, zebrafish embryos were incubated with ABC solution of the Vectastain elite ABC kit (Vector Laboratories, CA, USA) and, subsequently, with freshly prepared DAB (3,39-diaminobenzidine, Sigma-Aldrich, MI, USA) solution according to manufacturer’s instructions. Then, embryos were transferred to 90% glycerol through graded glycerol/PBS solutions (25%, 50%, 90%), laterally mounted and imaged using an Axio Imager M1 microscope (Zeiss, Oberkochen, Germany). We analyzed 20 larvae per group for each replicate, and data are expressed as mean ± SEM (n = 3 replicates). Statistically significant differences were assessed by one-way ANOVA with Dunnett’s post hoc correction.

### 2.10. Western Blotting

Zebrafish embryos at 24 hpf were anesthetized with tricaine MS-222 (Sigma-Aldrich, MI, USA) and dry stored at −80 °C. For protein extraction, embryos were defrosted on ice and homogenized in lysis buffer 1X containing a cocktail of protease inhibitors. Homogenates were diluted in a sample buffer (1:1 with 3.5% sodium dodecyl sulfate, 60 mM Tris, 10% glycerol and 0.25% bromophenol blue). The amount of the protein in the homogenates was quantified by the Bradford method (Bio-Rad, CA, USA). Proteins were separated by 10% polyacrylamide gel electrophoresis and transferred to nitrocellulose membranes (GE Healthcare, IL, USA). Membranes were saturated with 5% bovine serum albumin (BSA) in tris-buffered saline with Tween 20 (TBST 1X, 100 mM Tris, 150 mM NaCl, 0.1% Tween) for 1 h at RT under constant agitation to prevent non-specific binding. Primary antibodies (Onecut1 1:1000, Proteintech; β-actin 1:2000, Sigma-Aldrich, MI, USA), dissolved in 5% BSA diluted in TBST 1X, were applied for 24 h at 4 °C followed by the appropriate horseradish peroxidase-conjugated secondary antibodies (IgG goat anti-rabbit 1:5000, Sigma-Aldrich (MI, USA); IgG goat anti-mouse 1:5000, Sigma-Aldrich, MI, USA). After several washes in TBST, immunoblots were developed by chemiluminescence reaction using ECL detection reagent (Thermo Fisher, MA, USA). The density of bands was measured by Fiji software 1.51g and expressed as a fold change of the mean relative to controls (STD MO). Data are expressed as mean ± SEM (n = 3 replicates). Statistical differences were assessed by one-way ANOVA with Dunnett’s post hoc correction.

### 2.11. Tracking of Swimming Behavior

Swimming behavior was monitored using DanioVision instrument (Noldus Technology, Wageningen, The Netherlands). For locomotion tracking, single standard control and morphant larvae at 72 hpf were placed individually in 96-well plate in 100 μL of embryo medium. After an adaptation period of 10 min to a LED illumination source, larvae were subjected to a light stimulus of 100% white light intensity for 10 min, followed by a condition of darkness for another 10 min. The 10 min light/10 min dark period was repeated 2 times, for a trial duration of 50 min. Videos were tracking using the EthoVision XT software v. 11.0 (Noldus Technology, Wageningen, The Netherlands). Total distance moved and mean velocity were calculated for 12 larvae per group for each replicate.

### 2.12. Image Processing and Statistical Analysis

Raw images acquired from immunostainings (TUNEL and PH3), eye size and immunoblots were analyzed with Fiji software (version 1.51g) for quantitative analysis of apoptotic and proliferative cells, total eye area and densitometric band analysis, respectively. Image acquisition parameters were maintained equally for any experimental condition and for each replicate; figures were assembled using Photoshop (Adobe System Incorporated v. 20). All experiments were conducted in triplicate, except for RT-qPCR which was conducted in duplicate. Data analysis was performed using GraphPad Prism software v. 9.4.1 and data analysis was chosen based on statistical distribution (parametric and non-parametric). In detail, (i) statistical analysis of survival rate of controls (STD MO) and morphants injected with each *oc* MO was assessed by unpaired Student’s *t*-Test with Welch’s correction, comparing each *oc* MO vs STD MO, (ii) statistical significance of parametric data, was assessed by one-way ANOVA followed by Dunnett’s post hoc correction, (iii) statistical significance of non-parametric data was analyzed by Kruskal–Wallis with Dunn’s post hoc test. All data were expressed as mean ± standard error of the mean (SEM, * *p* ≤ 0.05, ** *p* ≤ 0.01, *** *p* ≤ 0.001, **** *p* ≤ 0.0001).

## 3. Results

### 3.1. Zebrafish Onecut Genes in Eye Development

In order to define the functional role of *Oc* gene family members and the evolutionary mechanisms responsible for vertebrate photoreceptors differentiation, we assayed the potential of *oc* genes in a teleost vertebrate occupying an intermediate phylogenetic position between non vertebrate chordates and mammals.

**Figure 2:**
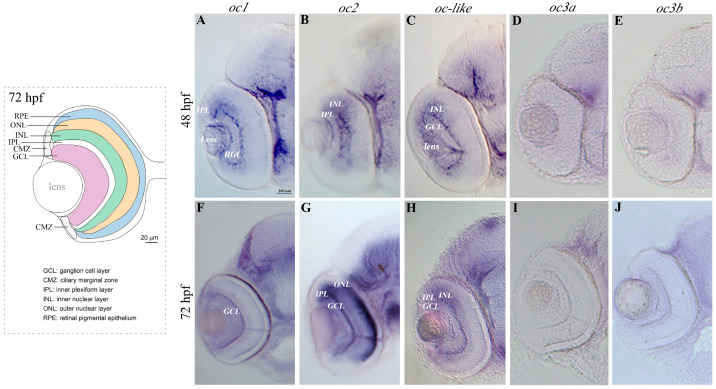
Territories of expression of *oc* genes in the zebrafish eye. On the left, schematic representation of the zebrafish eye section showing the retinal layers at 72 hpf. (**A**–**J**) In situ hybridization analysis of *oc1*, *oc2*, *oc-like*, *oc3a* and *oc3b* expression on zebrafish transverse eye sections at 48 hpf (**A**–**E**) and 72 hpf (**F**–**J**). *oc1* expression is localized in IPL and in RGC and remains in GCL at 72 hpf (**A**,**F**). *oc2* expression is observed in retinal IPL and INL at 48 hpf (**B**) and in GCL and ONL at 72 hpf (**G**), while *oc-like* transcript is reported in the INL and GCL at 48 hpf (**C**) and also in IPL at 72 hpf (**H**). No signal is observed for *oc3a* (**D**,**I**) and *oc3b* (**E**,**J**) in the zebrafish eye at both analyzed developmental stages. RGC, retinal ganglion cells; GCL, ganglion cell layer; INL, inner nuclear layer; IPL, inner plexiform layer; ONL, outer nuclear layer.

Five *oc* orthologous genes have been identified in the zebrafish genome, namely *oc1*, *oc2*, *oc-like*, *oc3a* and *oc3b*. Embryonic expression in the developing nervous system and retina of zebrafish embryos was previously reported for *oc1* and *oc-like*, but not for *oc2* and *oc3b*, and only partially for *oc3a* [31,32]. To understand which zebrafish *oc* genes are expressed during eye development, we used specific antisense RNA probes with low sequence homology with the other *oc* gene cDNAs (see Section 2). At 48 and 72 h post fertilization (hpf), *oc1*, *oc2* and *oc-like*, and not *oc3a* and *oc3b* are expressed in the developing eye (Figure 2A–J). In particular, *oc1* is expressed in the inner plexiform layer (IPL), the lens and the RGC at 48 hpf, only in the IPL at 72 hpf (Figure 2A,F); *oc2* is expressed in the ganglion cell layer (GCL) and in the IPL at 48 hpf, while at 72 hpf in the retina outer nuclear layer (ONL), in the IPL and in the PRCs (Figure 2B,G); *oc-like* is expressed at 48 hpf and at 72 hpf in the IPL (Figure 2C,H).

### 3.2. Functional Role of Onecut Genes in Eye Morphogenesis

**Figure 3:**
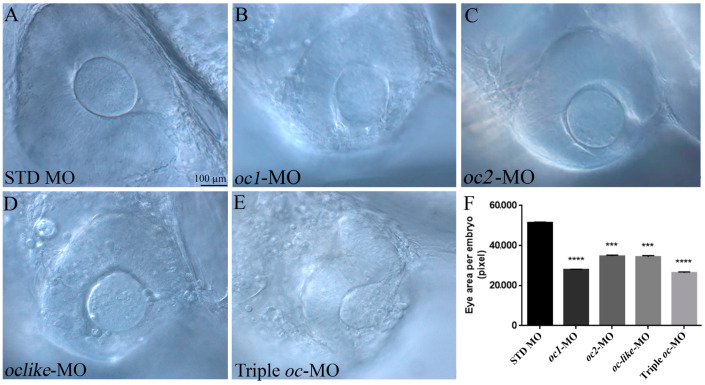
Eye phenotypes of *oc1, oc2, oc-like* and Triple morphant embryos. (**A**) Eye of control embryo injected with STD MO and (**B**–**E**) of embryos injected with *oc1*, *oc2*, *oc-like* and triple *oc*-MO. (**F**) Analysis of eye areas in *oc1*, *oc2*, *oc-like* and triple *oc*-MO embryos at 24 hpf. Data are expressed as mean ± SEM. Non parametric Kruskal–Wallis test with Dunn’s post hoc correction. *** *p* < 0.001, **** *p* < 0.0001.

To determine if *oc1*, *oc2* and *oc-like* genes are implicated in the development of retinal cell types, we knocked down the expression of single and multiple *oc* genes in zebrafish embryos by using the translational blocker antisense oligonucleotide morpholinos (MO). After dose dependent assays for each MO (Figure S1), and definition of their optimal concentrations to avoid differences of survival rate and unspecific alterations (Figures S1 and S2), the morphology of embryos microinjected with single and triple ATG-MOs was analyzed. They showed at 24 hpf a flat and distorted head with a rostral extension in comparison to control embryos injected with standard morpholino (STD MO) (Figure S2A–E). Reduced levels of *oc* activity caused microphthalmia, with 40–50% smaller eye diameter in comparison to the control (Figure 3A–F).

In support of phenotype specificity, we performed Western blot on protein extract from single and triple *oc*-MO and rescue experiments with *oc1* mRNA. Although the antibody recognizes all *oc* proteins, a significant reduction of *oc* proteins is visible in the western blot of the triple *oc*-MO (Figure S1D,E). The rescue experiments with the *oc1* mRNA, which showed to be the predominant factor among the three zebrafish *oc* expressed in retinal tissues, led to eye size recovery in embryos at 24 hpf (Figure S3).

**Figure 4:**
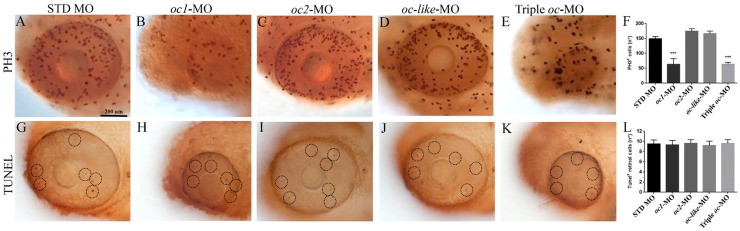
Cell proliferation and apoptosis assays on zebrafish *oc* morphant retinal cells. (**A**–**E**) Immunostaining for the mitotic marker PH3 in (**A**) Eye of control embryo injected with STD MO and (**B**–**E**) of embryos injected with *oc1*, *oc2*, *oc-like* and triple *oc*-MO. (**F**) Graphical analysis of proliferating cells in *oc1*, *oc2*, *oc-like* and triple *oc*-MO embryos at 24 hpf with respect to STD MO embryos. (**G**–**L**) Tunel assay showing no significant differences in the number of apoptotic cells (black circles) between control (**G**) and *oc1*, *oc2*, *oc-like* and triple *oc*-MO eyes (**H**–**K**). (**L**) Graphical analysis of the number of apoptotic cells observed in (**G**–**K**) retinas. Data are expressed as mean ± SEM. Non parametric Kruskal–Wallis test with Dunn’s post hoc correction. *** *p* < 0.001.

In order to investigate the cause(s) of the observed microphthalmia of zebrafish *oc1*/*oc2*/*oc-like* morphant embryos, we assayed proliferation and apoptosis levels in *oc* morphant eyes (72 hpf). Whole mount immunocytochemistry was performed to label mitotic cells with an antibody against PH3 [33,34] (Figure 4A–F). Quantitation of PH3-positive cells showed a significant reduction in the number of mitotic cells in *oc1* and triple morphants. On the other hand, no *oc* morphant embryos showed significant difference in the number of TUNEL^+^ cells compared with control retina (Figure 4G–L).

**Figure 5:**
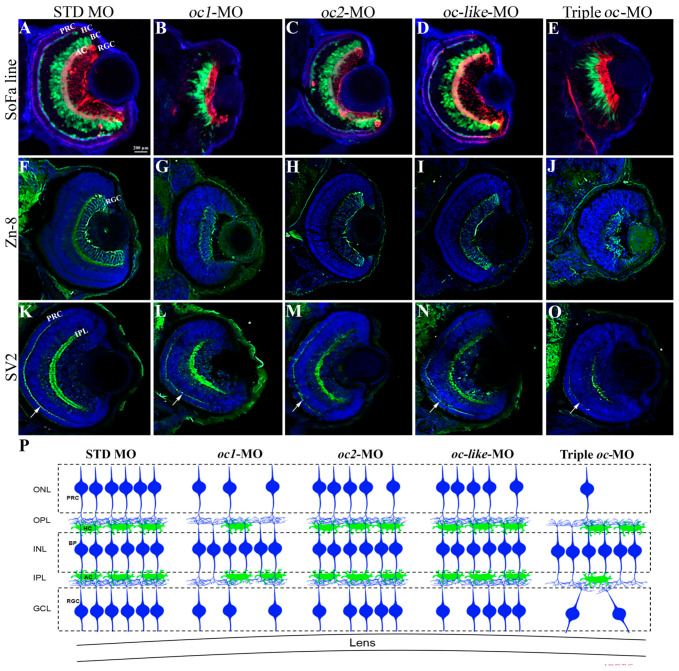
Effect of *oc* genes depletion on zebrafish retinal organization. (**A**–**E**) Sagittal sections of zebrafish retinas at 72 hpf from the SoFa1 transgenic line. (**F**–**J**) Immunostaining for Zn-8 in RGCs, and (**K**–**O**) for SV2 in IPL and PRC axon terminus (white arrows) of zebrafish eye at 72 hpf. (**A**,**F**,**K**) STD MO, (**B**,**G**,**L**) *oc1*, (**C**,**H**,**M**) *oc2*, (**D**,**I**,**N**) *oc-like* and (**E**,**J**,**O**) triple *oc*-MO eye sections. Retinal populations are unaltered following STD MO injection (**A**,**F**,**K**). A reduction of RGC (**G**) and AC (**B**) is reported in *oc1*-MO retina, and milder alterations are visible in *oc-like*-MO (**D**,**I**,**N**) and *oc2*-MO (**C**,**H**,**M**) respectively. Triple *oc*-MO retina (**E**,**J**,**O**) shows severe alterations and reductions in the RGC and a complete absence of PRC axon terminals (white arrow). (**P**) Schematic representation of the retinal alterations. The reduced number of cells represented in this schematic drawing is just an illustration and is not quantitative. AC, amacrine cells; BP, bipolar cells, HC, horizontal cells; PRC, photoreceptor cells; RGC, retinal ganglion cells; GCL, ganglion cell layer; IPL, inner plexiform layer; INL, inner nuclear layer; OPL, outer plexiform layer; ONL, outer nuclear layer.

To examine which retinal cell types were mainly affected by *oc* gene knockdown, we used Spectrum of Fates (SoFa1), a transgenic line carrying a combination of fluorescent proteins tagged to fate-specific promoters that allow simultaneous imaging of the main eye neuronal cell fates [35]. At 72 hpf, *oc1* and triple *oc-* MO-injected embryo (morphants) eyes were clearly abnormal compared with the STD MO controls (Figure 5A,B,E), while *oc2* and *oc-like* morphant eyes showed relatively moderate defects (Figure 5C,D). A strong reduction of RGC, AC and of the ONL, which contains PRCs and HCs, was observed in *oc1* and triple *oc* depleted embryos (Figure 5B,E). For further characterization of the cell layers in the morphant embryos we used two retinal cell type-specific markers, Zn-8 and SV2. In particular, Zn-8 labels the Alcam/Neurolin/DM-GRASP of RGCs (Figure 5F–J) and SV2 recognizes synaptic vesicles of the inner plexiform layer (IPL) and PRC axon terminus (Figure 5K–O). Retina of *oc1* and triple *oc* morphants exhibits high degree of RGC loss (Figure 5F,G,J) and full absence of PRC axon terminals in Triple ***oc***-MO embryos (Figure 5K,O). In Figure 5P, we schematically summarized the retinal alterations observed in the different *oc* morphant embryos. The almost complete absence of PRCs in Triple *oc*-MO is consistent with observations in *C. robusta*, where OCW transgenic embryos lack PRC [9]. It is worth noting that PRC differentiate in mouse double *oc1/ oc2*^−/−^ mutants, in which RGCs, but not PRCs, are reduced in number [11].

**Figure 6:**
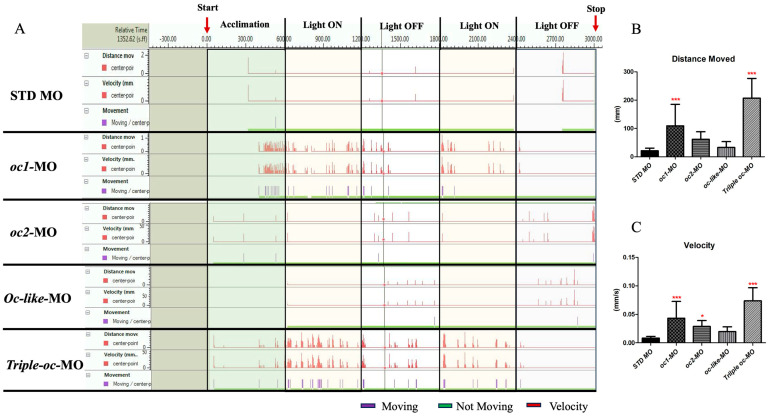
Alteration of the swimming activity in *oc* morphants. (**A**) Schematic representation of swimming activity of morphant larvae during light/dark stimuli. The recording traces show the increase in locomotion in *oc1*-MO and triple *oc*-MO larvae compared to *oc2, oc-like*-MO and STD MO injected larvae. (**B**,**C**) The graphs show the total distance moved (**B**) and the mean velocity (**C**), obtained by combining the values from 12 larvae per group. Statistical analysis was performed using the Kruskal–Wallis test followed by Dunn’s multiple comparison test. Bars represent mean ± SEM. * *p* < 0.05, *** *p* < 0.001.

To investigate the effects of retinal cell loss, we performed a behavioral analysis of larval locomotion in ***oc*** morphants and STD MO control larvae using the Noldus DanioVision system. Control and morphant larvae were placed in a 96-well plate and subjected to visual stimulation with two alternating cycles of light and dark, each lasting 10 min, as described in Section 2. Larval behavior in response to the light/dark cycles was recorded, and recording traces were generated by the EthoVision software v. 11.0 (Figure 6A and Figure S4). We observed an increase in larval movement in the dark versus light conditions, more pronounced in *oc1*-MO and in Triple-*oc-MO* compared to *oc2*-MO, *oc-like*-MO and STD MO, as shown by the red peaks in the recording trace (Figure 6A). Moreover, the total distance moved, as well as the velocity, were increased in *oc1*-MO and Triple *oc*-MO larvae compared to the STD, *oc2*, and *oc-like* morphant embryos (Figure 6B,C).

### 3.3. Onecut Target Genes in Eye Development

**Figure 7:**
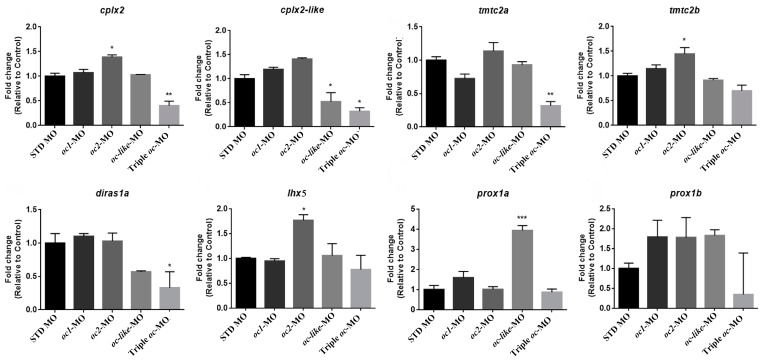
Altered expression of the zebrafish orthologs to *Ciona* DEGs. Altered levels of *cplx2*, *cplx2-like*, *tmtc2a*, *tmtc2b*, *diras1a*, *lhx5*, *prox1a* and *prox1b* transcripts in *oc* morphant embryos at 48 hpf by RT-qPCR. Gene expression was normalized to *rpl13a* expression. Data are expressed as mean ± SEM. (n = 2 for each study group). One-way ANOVA with Dunnett’s post hoc correction. * *p* < 0.05, ** *p* < 0.01, *** *p*< 0.001.

To investigate whether the zebrafish *oc1*, *oc2* and *oc-like* genes share a common and conserved pathway with other chordates, we examined whether the candidate *oc* target genes identified through RNA-seq analyses in ascidians [9] are also part of the genetic mechanism operating in zebrafish. We interrogated *oc* morphant embryos for the expression of the *Tmtc2*, *Diras*, *Cplx2*, *Lhx5* and *Prox1* orthologues. Since the correct identification of functional orthologous genes is critical in gene annotation to extrapolate functions across species barriers, we analyzed each zebrafish ortholog/paralog of *Tmtc2* (*tmtc2a*, *tmtc2b*), *Diras* (*diras1a*, *diras1b*), *Cplx2* (*cplx2*, *cplx2-like*), *Prox1* (*prox1a*, *prox1b*) and *Lhx5* (*lhx5*) by RT-qPCR. Expression of *tmtc2a*, *diras1a*, *diras1b* and *cplx2-like* genes has already been documented in zebrafish eye development [31,36,37]. Following the individual downregulation of *oc* genes, we observed upregulation of *cplx2*, *tmtc2b* and *lhx5* following ***oc2*** depletion; *cplx2-like* decreased and *prox1a* increased in *oc-like* morphant embryos; no significant changes were detected in *oc1* morphants (Figure 7). Remarkably, expression levels of *cplx2*, *cplx2-like, tmtc2a* and *diras1a* were strongly reduced in triple *oc* morphants (Figure 7). As to the latter, these results suggest the hypothesis of a combinatorial action of *oc* genes in their function during eye development in zebrafish.

## 4. Discussion

From an evolutionary perspective, we investigated the extent of *Oc* gene functional conservation in retinal differentiation, by assessing their potential in inducing retinal cell types differentiation in a teleost fish. Our work illustrates that tracing components of the *oc* gene regulatory network can provide a framework for understanding how complex traits originate during evolution.

Despite the extra gene duplication of *oc* genes, zebrafish embryos represent an excellent model to underscore the full function of these genes during retinal differentiation. Only three of the five *oc* genes are expressed in retinal cell populations and the experimental approaches that this model offers clearly provide advantages in assessing the functional roles of these genes during formation of different eye cell types.

The analysis of the retinal phenotypes in knockdown zebrafish embryos suggests a genetic redundancy and an evolutionarily conserved role of *oc* genes in different retinal tissue types formation and differentiation.

*Oc* gene redundancy has already been noticed comparing mouse single and double *Oc* knockout models. Likewise, our demonstration that concomitant downregulation of zebrafish *oc1*, *oc2* and *oc-like* results in more severe phenotypes and the almost complete absence of photoreceptors, indicates that the three zebrafish orthologous genes are functionally redundant.

We highlight the evolutionarily conserved and redundant regulatory relationships of chordate *oc* genes [3,7]. In this view, our analyses also evidenced a primary functional role exerted by the *oc1* gene in retinal tissue types formation with respect to the other family members expressed in the developing eye.

The functional redundancy of *Oc* genes observed in both mouse and zebrafish vertebrate retinas suggests that their evolution did not follow the classical patterns of duplicated genes [23]. Therefore, the various *oc* gene copies seems to be not fully equivalent, and in both zebrafish and mice, it is the *Oc1* gene that exhibits greater functional efficacy than the other gene copies. According to the theory of stable redundancy proposed by Nowak and colleagues (1997) [38], this implies that the presence of all *Oc* genes ensures greater functionality for the proper development of retinal territories. Moreover, these genes were recruited during vertebrate evolution for the development of novel organs, such as the liver and pancreas. The presence of multiple regulatory elements, also called shadow enhancers, may contribute to the stability of *oc* genetic redundancy, increasing the robustness of the phenotype and providing a much more precise and consistent spatio-temporal expression profile [39]. Our study provided a comparative analysis of the functional role of *Oc* genes among chordates and highlighted their ancestral role in photoreceptor differentiation, which has been conserved in zebrafish, with all three *oc* genes expressed in the retina contributing to this process. In zebrafish, both the *oc1*-MO and triple *oc*-MO injections lead to a notable reduction of PRCs in the ONL. In support of this observation, altered visuomotor responses to light stimuli were observed (Figure 6), similar to those already seen in larvae lacking eyes [40]. Specifically, zebrafish *oc1* and triple morphant larvae exhibited similar elevated activity and increased, undirected light-seeking behavior, independent of the normal light on/off stimuli.

This phenotype is reminiscent of that observed in ascidian transgenic embryos carrying ectopic expression of a constitutive *oc* repressor protein [9]. Although ascidians lack a retina-like tissue and only possess one pigment cell and three groups of PRCs to control their shadow response activity, they share many components of the *oc* genetic pathway responsible for PRC differentiation with vertebrates [9]. In contrast, *Oc1*^−/−^ and *Oc2*^−/−^ double mouse mutants revealed a fundamental role played by these transcription factors in the formation of HC, RGC and AC, but did not show such a severe reduction in PRCs, where they have been selectively maintained for cone differentiation [11,17,21]. Therefore, two possible scenarios can explain the functional diversity of *Oc* genes in mouse photoreceptor development. First, the selective role in cone differentiation has been specifically acquired in mammals. A second hypothesis might be that mammalian photoreceptor differentiation are also controlled by the concomitant action of other genes. Further studies on other mammalian models will be necessary to further clarify this issue and to establish the mechanism underlying the functional evolution of *Oc* genes.

The small-eye phenotype observed in zebrafish *oc* morphants is the consequence of a reduced cellular proliferation in the various tissues of the retina, as it occurs in the *Oc1*^−/−^ and *Oc2*^−/−^ double knockout mouse model [11]. These results seem to reflect a conserved functional role among vertebrates of *Oc* genes in regulating the molecular components for the correct retinal tissue differentiation. Also, the *Oc* target genes appear to be conserved from non-vertebrate to vertebrate chordates. By analyzing the altered expression of *oc* target genes in zebrafish morphant embryos, as identified by differential RNAseq analyses in the ascidian *C. robusta* [9], we have demonstrated that zebrafish *oc* orthologues regulate a common set of downstream genes. These *Oc* target genes shared by *Ciona*, zebrafish and mice, are involved in synapse formation and the regulation of neurotransmission. In particular, *Cplx2* encodes a soluble protein involved in synaptic vesicle exocytosis (reviewed in [41]), and in mice, it plays a role in ON-bipolar neurons neurotransmission [41,42,43]. Diras, a small GTPase Ras member, regulates neuronal functions, including neurotransmission, as well as neuron migration and proliferation [36,44]. Tmtc2 encodes an endoplasmic reticulum adapter protein involved in calcium homeostasis [45]. In humans, Diras3 and Tmtc2 are implicated in the onset of glaucoma, a progressive optic neuropathy caused by the loss of RGCs, further confirming the important role of *Oc* proteins and their target genes in the onset of human diseases [45,46,47].

## 5. Conclusions

In this study, we demonstrate an evolutionary history of collaboration among *oc* genes in driving various aspects of retinal development and PRC differentiation. Our work contributes to expanding our understanding of the *Oc* genetic pathway and provides evidence of its conservation during chordate evolution, from invertebrate tunicates to vertebrates. Furthermore, the conserved *Oc* target genes are involved in synaptic neurotransmission and retinal diseases, and their connection to the *Oc* genetic cascade could provide important insights for future studies on their neural activity and role in related pathologies.

## Data Availability

All data and related information are available upon request to annamaria.locascio@szn.it.

## Supplementary Materials

The following supporting information can be downloaded at: https://www.mdpi.com/article/10.3390/cells13242071/s1, Figure S1: Survival of zebrafish *oc*-MO embryos; Figure S2: Morphology of zebrafish oc morphant embryos at 24 hpf; Figure S3: Rescue experiment of zebrafish oc1 morphant phenotype; Figure S4: Swimming behavior of oc morphants; Table S1: Primers used for PCR and RT-qPCR amplification, sequences of morpholino oligonucleotides used for knockdown experiments.
