# Supplementary material for: The Zebrafish Retina and the Evolution of the Onecut-Mediated Pathway in Cell Type Differentiation
Source: Cells. 2024 Dec 15;13(24):2071. doi: 10.3390/cells13242071 (PMC11675081; doi:10.3390/cells13242071)
Supplement: Supplementary file 1 [file cells-13-02071-s001.zip › cells-3167577-supplementary.pdf]

Zebrafish gene name	Oligo Fw	Oligo Rev	Probe length
oc1	5′-GCACACCGACACCATGCACTGCTG-3′	5′- GATGACGTTGATGGGCACCATGC-3′	928 bp
oc2	5′-GGATGCGTACGCCATGAACCCAG-3′	5′- GATCTCTTCCAGCTGCCCGGAGC-3′	832 bp
oc-like	5′CAGCACAGGTTAAGCTCTGCAGCTCC-3′	5′- GCGCATTATGTATGATCGTCGTACAG-3′	1259 bp
oc3a	5′-AACTCTGTCGGATCTCCTGC-3′	5′-GTGCTGCTCCTGTTCCTTTC-3′	1220 bp
oc3b	5′-GAACTCTGTCGGACCTGCT-3′	5′-TCCTGCTCCTTTCTCTTGCA-3′	1200 bp
cplx2a	5’-GAACAGAACTCGGACATAGTGTAC-3’	5’-CAATCTTGCTGTGATCACAGAAC-3’	1691 bp
cplx2-like	5’-TCCTGCTGAAGGCAGCTATGG-3’	5’-CCTCATGGCTCTAGATCAGAG-3’	1133 bp
tmtc2a	5’-TCACTAGAGCGCTTACATTCCTG-3’<br>5’-CATTCCTGTACCTGCCATCTG-3’	5’-GATCCAGCTCTATCGCCTTCAG-3’<br>5’-GAAGTAACGCTCTGCTTCAGAC-3’	1289 pb
tmtc2b	5’-CTAGTATGGGCTACTGTCTGC-3’<br>5’-TCCTCTAGAACGTTGGTGTTGG3’	5’-TTCTTCAGTGTCCAGCTGCGC-3’<br>5’-CTGACCATAATGCATGTAGCAG-3’	810 bp
diras1a	5’-CGCATCTATGGTCGCATCAC-3’	5’-GGAAACATAGACACCGACAG-3’	1349 bp
diras1b	5’-GAGCAGAGCAACGACTATCG-3’	5’-TCCTGGAAGAGCTCAGTGAC-3’<br>5’-GATTAGTCTTAGCCGAGGTC-3’	439 bp

RT-qPCR gene name	Oligo Fw	Oligo Rev
cplx2a	5’-ACAACAAGAGGAAGAGCGCA-3’	5’-TTTTGGGGCGATCAAGGAG-3’
cplx2-like	5’-TCGAGCACCAGGACTGAAAC-3’	5’-GTTTGGCCTTCCTCTCCTCC-3’
tmtc2a	5’-AAGAGGCAGTTCGGAAATG-3’	5’-TGGGCAGGAATATGATCTGG-3’
tmtc2b	5’-TGTGACTTGGCTGAGATCTC-3’	5’-CCTAGGCATCTTCTGTATCGCT-3’
diras1a	5’-TCGGATCTCCTAATGCTTCC-3’	5’- AGACCACCACTCGGTAATCG-3’
elfa1	5’-ACCTACCCTCCTCTTGGTCG-3’	5’-GGAACGGTGTGATTGAGGGA-3’
rpl13a	5’-GAAGATGGCGGAGGGGCA-3’	5’-CCCTCACAGCGTACAACCA-3’

Gene name	Morpholino Oligo
oc1	5’-GCTTGTGGACAAATCATCTATCTGA-3’
oc2	5’-TAGGCGTTATAGGCAGTCTTCATTC-3’
oc-like	5’-ACATCTCTCCCATATTACCATCCAT-3’

Table S1. Primers used for PCR and RT-qPCR amplifications; sequences of morpholino oligonucleotides used for knockdown experiments

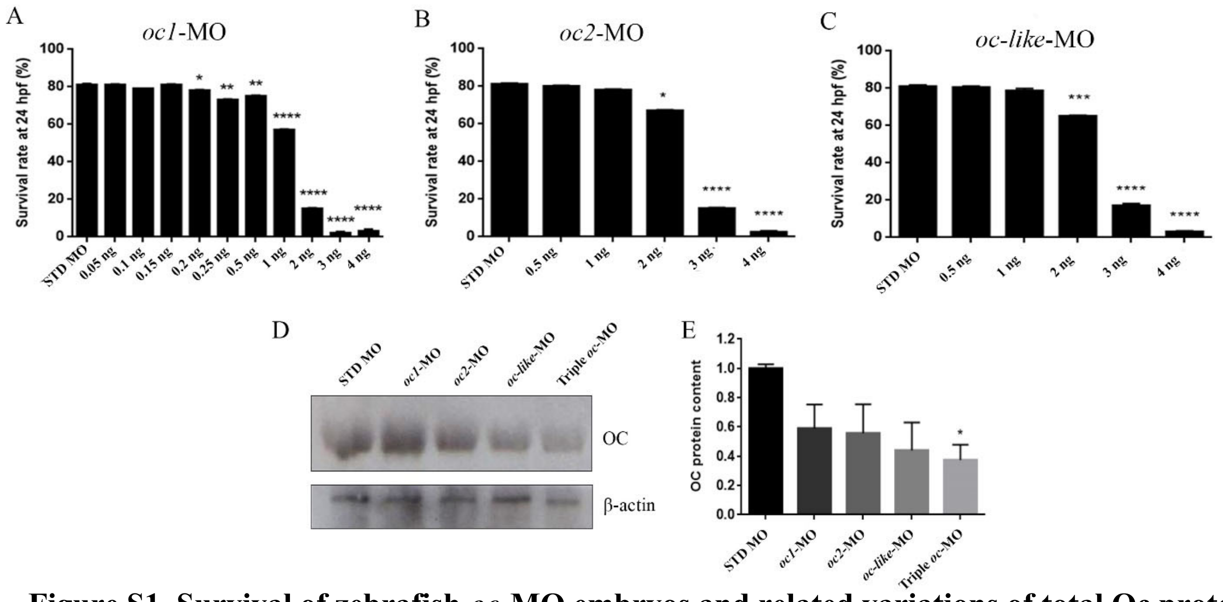

Figure S1. Survival of zebrafish *oc*-MO embryos and related variations of total Oc proteins.
(A-C) Diagram of the survival rate of zebrafish embryos at 24 h post fertilization following microinjection of *oc1*, *oc2*, *oc-like*, and STD MO. Data are expressed as mean ± SEM (n=3 for each study group). Significant differences of survival rate were analysed by unpaired Student's t-test with Welch's correction (* $p < 0.05$; ** $p < 0.01$; *** $p < 0.001$; **** $p < 0.0001$). (D) Western blotting and (E) densitometry of Oc protein in *oc1*, *oc2*, *oc-like* and, triple *oc*-MO. Data are expressed as mean ± SEM. (n=3 for each study group). One-way ANOVA with Dunnett's *post hoc* correction, * $p < 0.05$.

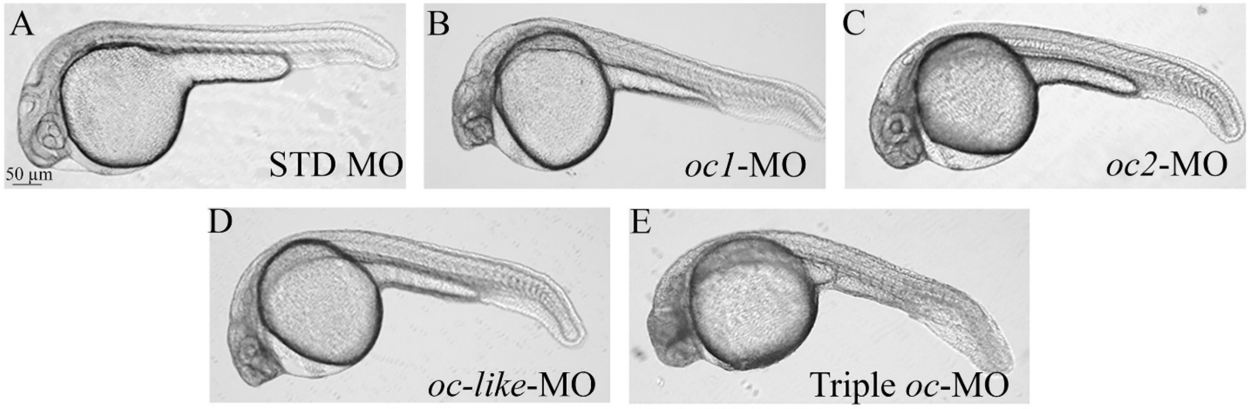

Figure S2. **Morphology of zebrafish *oc* morphant embryos at 24 hpf**.
(A) Control embryo injected with STD MO. (B) *oc1* embryo injected with 300 pg of morpholino. (C, D) *oc2* and *oc-like* embryos injected with 500 pg of the corresponding morpholino. (E) Triple *oc* morphant embryo injected with 150 pg of *oc1* and 250 pg of *oc2* and *oc-like* morpholinos. Anterior head alterations and eye size reduction are particularly evident in *oc1* and triple *oc*-MO. Lateral view of embryos, anterior is on the left.

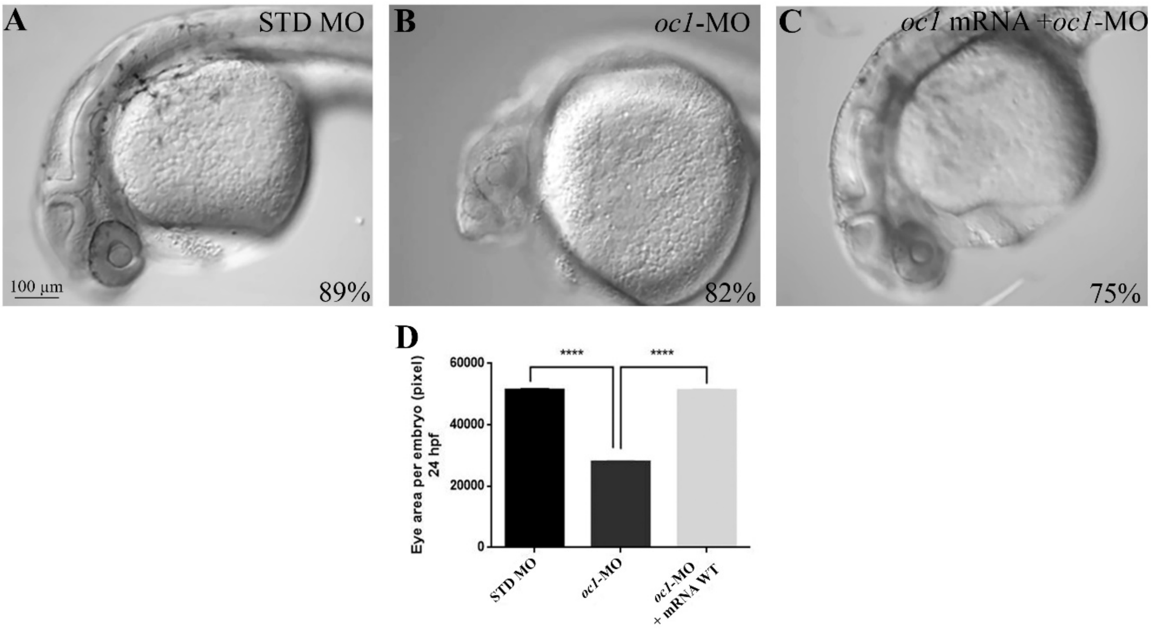

Figure S3. **Rescue experiment of zebrafish *oc1* morphant phenotype**.
(A) Control embryo at 24 hpf injected with the STD MO. (B) *oc1* morphant embryo at the same stage injected with 300 pg of *oc1*-MO. (C) Embryo injected with 300 pg of *oc1*-MO and 200 pg of *oc1* mRNA. Lateral view of all embryos, anterior is on the left. The percentage reported for each phenotype was calculated on the total number of live embryos obtained from injection in triplicate. (D) Analysis of eye areas in *oc1*-MO and *oc1*-MO+*oc1* mRNA. Data are expressed as mean ± SEM. Non parametric *Kruskal-Wallis* test with Dunn's *post hoc* correction. ***p<0.001, ****p<0.0001, ** <0.01.

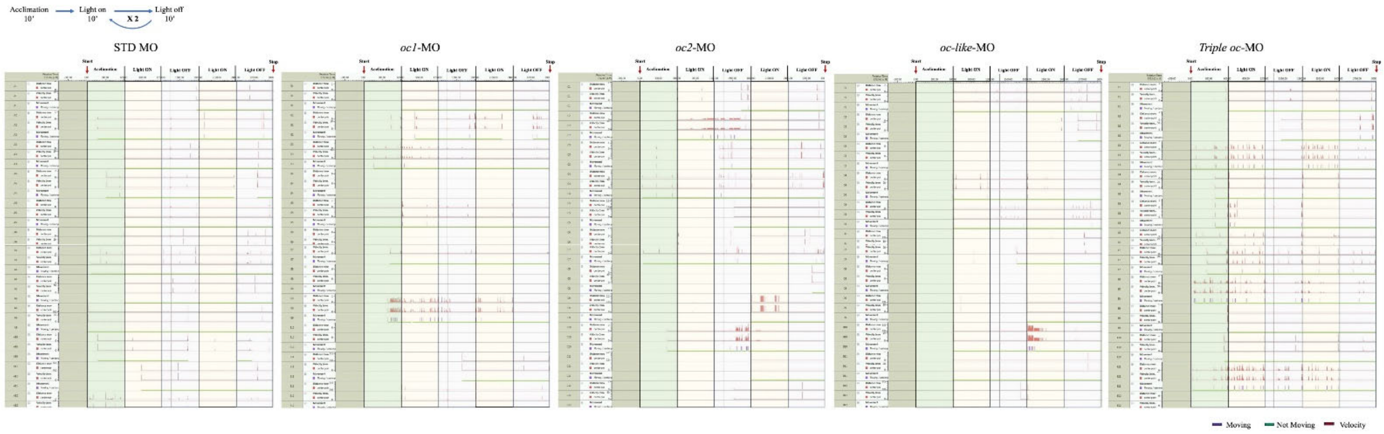

Figure S4. Swimming behaviour of *oc* morphants. Schematic representation of the entire locomotory activity of morphant larvae subjected to a 2 alternating cycles of light/dark stimuli for 10 min, and for a trial duration of 50 min. Red and purple peaks and green lines represent the movement/not movement recorded by 12 larvae each group.
